# Epidemiology of Metabolic Dysfunction–Associated Steatotic Liver Disease in South Asian Ethnicities: A Systematic Review and Meta‐Analysis

**DOI:** 10.1111/jgh.70080

**Published:** 2025-09-26

**Authors:** Michael James, Melanie Smuk, Naheeda Rahman, Georgia Black, William Alazawi

**Affiliations:** ^1^ Centre for Immunobiology, Blizard Institute Queen Mary University of London London UK; ^2^ Centre for Genomics and Child Health, Blizard Institute Queen Mary University of London London UK; ^3^ Centre of Prevention, Detection and Diagnosis, Wolfson Institute of Population Health Queen Mary University of London London UK

**Keywords:** ethnicity, MASLD, metabolic, south Asian

## Abstract

**Background and Aims:**

People of South Asian ethnicities are at increased risk of metabolic syndrome, but the epidemiology of metabolic dysfunction–associated steatotic liver disease (MASLD) in this population remains poorly understood. This systematic review and meta‐analysis assesses the prevalence, cross‐sectional severity, and clinically relevant endpoints of MASLD in this group.

**Methods:**

Studies from January 2002 to November 2023 across PubMed, Embase, Ovid, and Cochrane databases, meeting inclusion criteria, were included. We performed a random effects meta‐analysis to calculate pooled prevalence and proportions.

**Results:**

Sixty‐two articles met inclusion criteria (*n* = 40 745). Fifty‐three were based in India, 4 in Sri Lanka, 3 in Pakistan, and 2 in Bangladesh. The pooled prevalence of MASLD in studies that did not select for T2DM status was 34.7% (95% CI 29.0%–40.5%, *I* ^2^ = 99.5%) and higher in studies with only T2DM patients, 60.0% (95% CI 48.9%–71.1%, *I* ^2^ = 99.0%). The pooled prevalence was higher in urban (47.1%; 95% CI 36.1%–58.1%, *I* ^2^ = 99.7%) compared with rural (18.5%; 95% CI 13.7%–23.3%, *I* ^2^ = 96.2%) settings. The pooled average proportion of participants with advanced fibrosis (≥ F3) was 14.0% (95% CI 9.2%–18.7%, *I* ^2^ = 91.5%). Four studies reported on clinical outcomes of interest; however, all had different endpoints, and so meta‐analysis was not possible.

**Conclusions:**

MASLD prevalence in South Asian ethnicities is high, and the proportion of people with advanced fibrosis may be higher compared with multiethnic populations, though high‐quality longitudinal studies are needed to better understand the epidemiology of this cohort.

**Abbreviations**
ALT: alanine aminotransferase
APRI: aspartate aminotransferase/platelet ratio index
AST: aspartate aminotransferase
BMI: body mass index
CI: confidence interval
ELF: enhance liver fibrosis score
FIB4: fibrosis 4 score
HCC: hepatocellular carcinoma
MASH: metabolic dysfunction–associated steatohepatitis
MASLD: metabolic dysfunction–associated steatotic liver disease
MRI‐PDFF: magnetic resonance imaging proton density fat fraction
NAFLD: nonalcoholic fatty liver disease
NAS: nonalcoholic fatty liver disease activity score
NASH: nonalcoholic steatohepatitis
NIH: National Institutes of Health
PCOS: polycystic ovarian syndrome
PRISMA: preferred reporting items for systematic review and meta‐analysis
RCT: randomized control trial
T2DM: Type 2 diabetes mellitus
VCTE: vibration‐controlled transient elastography

## 1 Introduction

Metabolic dysfunction–associated steatotic liver disease (MASLD), previously referred to as nonalcoholic fatty liver disease (NAFLD), affects approximately 25%–30% of the general population [1, 2] and is the hepatic manifestation of the metabolic syndrome. It is a spectrum, ranging from simple steatosis through hepatocyte injury and the progressive inflammatory form, metabolic dysfunction–associated steatohepatitis (MASH), which can lead to fibrosis in 25%–40% of these patients [3]. At the severe end of this spectrum is MASH‐related cirrhosis, decompensation, and the development of hepatocellular carcinoma (HCC), all of which have a substantial impact on health‐related quality of life [4], morbidity, and mortality [5], with the greatest socioeconomic burden of MASLD associated with the late stages of the disease [6].

Coexisting Type 2 diabetes (T2DM) has been identified as one of the strongest independent predictors of progression to advanced liver disease [7], with a global prevalence of MASLD in the T2DM population estimated at 65% [8]. Significant ethnic disparities in prevalence and severity of MASLD have been identified, with the majority of data based on populations from the United States (US) [9, 10]. In a systematic review and meta‐analysis, Rich et al. [11] showed the highest prevalence in people of Hispanic ethnicity, with an increased incidence of MASH compared with White and African American populations. However, there were too few people of Asian ethnicity (including both South and East Asian) to be included in the final meta‐analysis.

People of South Asian ethnicities with MASLD may represent a distinct cohort. There is an increased risk of MASLD and T2DM, with Bangladeshi ethnicity being an independent risk factor for MASLD development [12]. There is a potentially more aggressive phenotype of disease, with fibrosis occurring a decade younger than in Caucasian people with comparable stage and severity of liver injury. We have previously shown that noninvasive tests, derived in largely White populations, are less accurate in people of South Asian ethnicities [13]. These disparities are complex and multifactorial, yet the overall prevalence, severity, and liver and nonliver related outcomes of South Asian patients with MASLD are poorly understood, despite the fact that approximately a quarter of the world's population lives in South Asian countries, with a much wider global diaspora [14]. Understanding the epidemiology and severity of disease in South Asian, as well as other, ethnicities is essential to understand the biology of disease, develop new therapies, and design care pathways that are relevant to individual populations. The aim of this systematic review and meta‐analysis is to characterize the global prevalence, cross‐sectional severity, and clinical outcomes of people of South Asian ethnicities with MASLD.

## 2 Methods

### 2.1 Nomenclature

We report our findings using the new consensus nomenclature [15] as we believe this change best describes the disease's close relationship with other aspects of the metabolic syndrome. However, given this is a recent development, most included studies within the timeframe specified were conducted under the old definitions of NAFLD and NASH. In order to maximize our data capture, we undertook searches using both NAFLD/NASH and MASLD/MASH terms. We have justified the inclusion of both in our meta‐analysis based on the high levels of overlap between patients previously classified as having NAFLD using historical criteria, compared with the new MASLD criteria [16, 17, 18].

### 2.2 Literature Search Strategy

This review was conducted in adherence with the Preferred Reporting Items for Systematic Review and Meta‐analysis (PRISMA) [19] and the Meta‐analysis of Observational Studies in Epidemiology (MOOSE) [20] guidelines and was registered on the International Prospective Register of Systematic Reviews (PROSPERO). We searched PubMed, Embase, Ovid, and Cochrane databases from January 2002 to November 2023 using the search terms “non‐alcoholic fatty liver disease (NAFLD),” “metabolic dysfunction associated steatotic liver disease,” “MASLD,” “fatty liver,” “Non‐alcoholic steatohepatitis,” “NASH,” “metabolic dysfunction associated steatohepatitis,” “MASH,” “prevalence,” “severity,” “epidemiology,” “risk score,” “complications,” “decompensation,” “cirrhosis,” “hepatocellular carcinoma,” “HCC,” “death,” “South Asian,” “India,” “Pakistan,” “Sri Lanka,” and “Bangladesh” using combinations of Boolean operators. The full list of search terms and combinations can be found in the Supporting Information: methods section (Figure S1).

### 2.3 Eligibility Criteria

Inclusion criteria for studies were as follows: (1) studies involving adult participants, age 18 or above; (2) participants had a diagnosis of MASLD/MASH based on radiological, histological, or other noninvasive tests such as vibration controlled transient elastography (VCTE) or underwent risk stratification with Fibrosis 4 (FIB4) score, enhanced liver fibrosis (ELF) test, or aspartate aminotransferase (AST)/Platelet Ratio Index (APRI); (3) were cohort, cross‐sectional, case‐control studies, or randomized control trials (RCTs) (N.B. relevant case‐control studies were included in meta‐analysis of cross‐sectional severity only); (4) study populations included a cohort of South Asian participants (defined as South Asian, Indian, Pakistani, Bangladeshi, Sri Lankan), with or without another comparator ethnicity cohort; (5) reported on at least one of prevalence, cross‐sectional severity (measured by histological fibrosis score), or complications such as hepatic decompensation (specifically incidence of new ascites, encephalopathy, or variceal bleed), incidence of HCC, or death.

Studies were excluded if they (1) included participants with other aetiologies of liver disease in a pooled analysis; (2) diagnosed MASLD based solely on liver enzymes given their poor correlation with the presence of MASLD [3]; (3) did not have data on South Asian participants and/or did not stratify outcomes based on ethnicity; (4) were not in the English language; (5) were case reports, letters, conference abstracts/posters, or did not contain original data (e.g., review articles); (6) did not report on prevalence, severity, or the clinical outcomes previously mentioned; (7) reported on patient cohorts that were judged to be too distinct and overselected from a general and diabetic population as to be representative.

### 2.4 Study Selection and Data Extraction

Studies' titles and abstracts were screened for relevance after duplicates were removed by automation tools (study type and date range) in EndNote version 20 (MJ). This was followed by an independent full‐text review by two investigators (MJ and NR) to assess studies for inclusion. If any disagreement regarding study inclusion arose, this was resolved by a third investigator (WA). Data on participant demographics (age, sex, ethnicity, and body mass index [BMI]), country/administrative region, rural/urban population based on the description within the study if available, community/hospital based study (primary/secondary care), method of MASLD diagnosis, MASLD prevalence, prevalence of T2DM, MASLD severity (based on histological scores), and data on clinical incidence of endpoints (hepatic decompensating events, HCC, and death) were extracted. Study quality was assessed using the National Institutes of Health (NIH) Quality Assessment Tools for cross‐sectional/observational, cohort studies, and case‐control studies [21]. We chose not to exclude papers based on quality score alone, given the ill‐defined thresholds for assessing this.

### 2.5 Data Synthesis and Statistical Analysis

All data analysis was performed using Stata version 17. A random effects meta‐analysis was conducted to explore pooled estimates over multiple studies. Pooled estimates with 95% confidence intervals (CIs) were created for study mean age and study mean BMI. We distinguished between studies that only included people with T2DM (the T2DM subgroup) and those that did not select participants based on T2DM status.

The pooled prevalence with 95% CI for all participants, and separately for males and females, was calculated for studies involving the unselected general population and then for the T2DM subgroup. The pooled prevalence of MASLD based on the method of diagnosis, study setting (hospital versus community), and geographic location was also calculated. Cross‐sectional severity of steatosis and fibrosis was analyzed by calculating pooled percentages. For steatosis, cross‐sectional pooled percentages of study participants with S1–S3 steatosis on ultrasound were calculated for studies that did not select based on T2DM status and for the T2DM subgroup of studies. Similarly, for fibrosis, cross‐sectional pooled proportions and percentages for NAFLD activity score (NAS) F0–F4 fibrosis on biopsy were calculated for the studies that did not select based on T2DM status and the T2DM subgroup of studies.

There were too few studies with data on complications previously described in participants of South Asian ethnicities to perform a meta‐analysis. The effect of interstudy heterogeneity on the meta‐analysis was evaluated using the inconsistency index (*I* ^2^). A cut‐off of > 75% *I* ^2^ was used to indicate a high level of heterogeneity [22]. Because of high levels noted between included studies, a leave‐one‐out sensitivity analysis was performed, where studies were removed one at a time to assess for undue influence on the overall pooled estimate. The influence of a single study was found in one meta‐analysis only and is reported on in the results.

We assessed publication bias in our meta‐analyses via Egger's test and assessed the impact of variables such as BMI and year of publication on prevalence using a meta‐regression analysis. There was a paucity of studies that recorded age and sex together, and as such, we were unable to provide a meaningful meta‐regression analysis for these variables.

### 2.6 Quality Assessment of Included Studies

Quality assessment of studies was undertaken using the NIH Quality Assessment Tools for cross‐sectional/observational, cohort studies, and case‐control studies [21]. Scoring of studies was calculated based on whether they fulfilled the criteria detailed in the appropriate tool, with a total score of 14 available for cross‐sectional and observational cohort studies and 13 for case‐control studies.

## 3 Results

### 3.1 Literature Search

**FIGURE 1:**
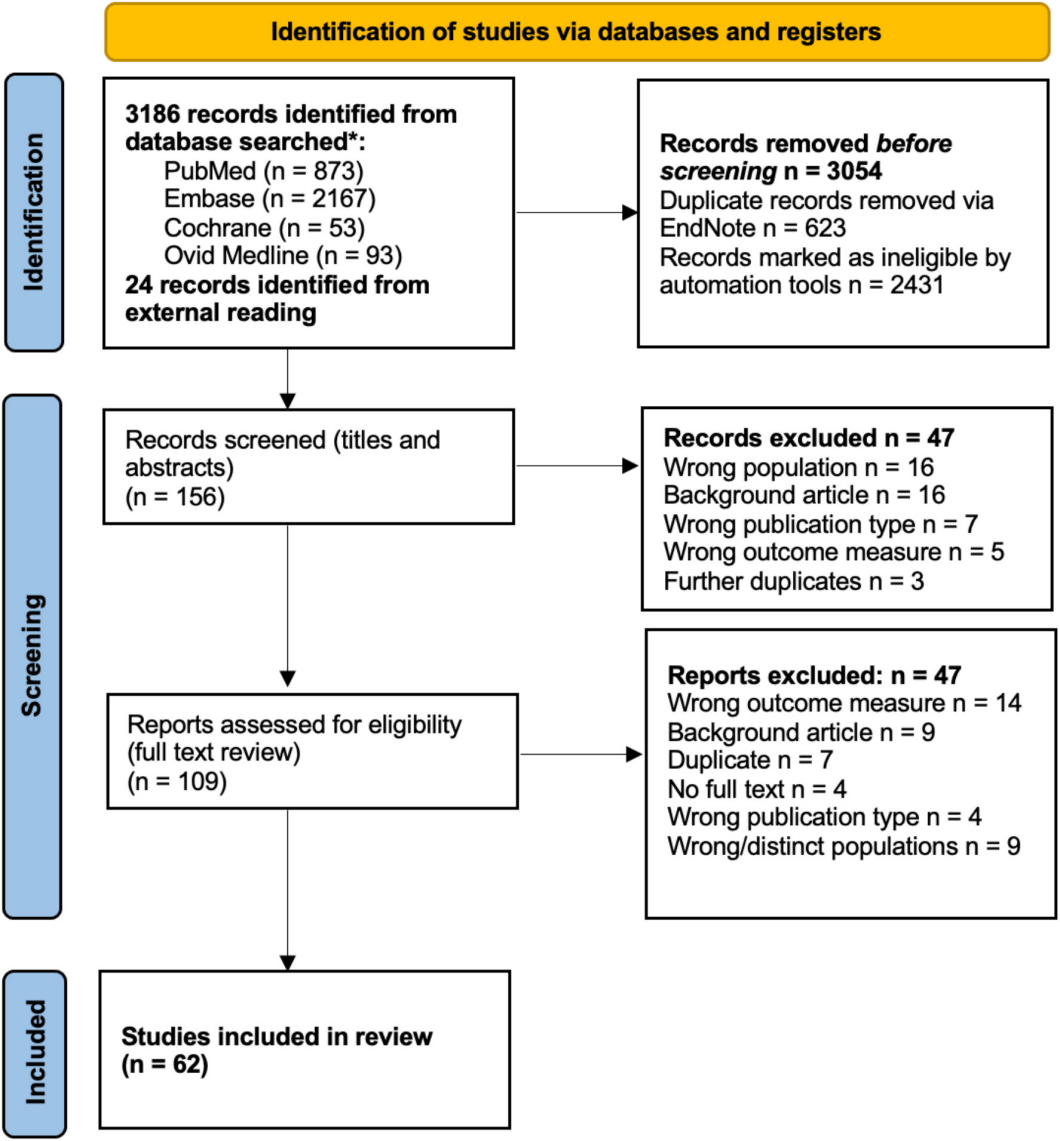
Preferred Reporting Items for Systematic Reviews and Meta‐Analyses (PRISMA) flow diagram of identification of studies. Of 3186 records identified in initial searches and 24 records identified in external reading, 62 were included in the final meta‐analysis after removal of duplicates, use of automation tools, screening of abstracts, and full‐text review.

The initial search strategy yielded 3186 articles from PubMed, Embase, Ovid, and Cochrane databases, with 24 additional records identified from external reading (see PRISMA diagram in Figure 1). After applying automation tools for study type and date range, and removing duplicates, the titles and abstracts of 156 potentially relevant articles were screened, and of these, 109 underwent a full‐text review. A total of 62 articles met inclusion criteria and were included in the final analysis. Of note, nine studies did not meet inclusion criteria and were not included in the meta‐analysis as their study populations were assessed to be too heavily selected and not representative: Three studied MASLD prevalence in patients with polycystic ovarian syndrome (PCOS) [23, 24, 25]; one studied patients admitted with acute myocardial infarction [26]; three studied patients who were morbidly obese, awaiting and undergoing bariatric surgery [27, 28, 29]; and two studied MASLD in pregnancy [30, 31]. On full‐text review, there was full agreement between the first and second reviewers with regard to article inclusion.

### 3.2 Study Characteristics

In the 62 studies included, there were a total of 40 745 participants (a breakdown of studies included is found in Table S1). Fifty‐three studies were based in India, 4 in Sri Lanka, 3 in Pakistan, and 2 in Bangladesh. From our searches, we were unable to identify any studies involving 1st or 2nd generation South Asian migrants set in other countries, reporting on this group as a distinct cohort in the analysis. Forty‐nine studies were undertaken in secondary care, most in hospital outpatient departments (*n* = 20 217 participants), and 12 studies were undertaken in the community (*n* = 18 370), with one study taking place in both primary and secondary care (*n* = 2158). Forty‐four studies reported on prevalence (*n* = 32 094), 33 of these reported on a South Asian population that did not select for participants with T2DM (*n* = 28 326), and 11 reported on the T2DM subgroup of studies (*n* = 3768). Sixteen studies reported stratification by ultrasound‐determined steatosis grades S1–3 (*n* = 7413), and 15 reported severity by fibrosis score F0–F4 (*n* = 9431). Only four studies were identified that assessed clinical outcomes in participants of South Asian ethnicities: one assessing the annual rate of HCC diagnosis [32], one assessing the incidence of hepatic decompensation [33], one assessing the mortality in participants of South Asian ethnicities and MASLD [34], and one assessing all three outcomes [35].

### 3.3 Participant and Population Demographics

The mean age of all participants in all studies with data available (*n* = 45 studies) was 46.3 years (95% CI 43.6–49.0, *I* ^2^ = 99.76%). Among the studies in the T2DM subgroup, the mean age of all participants was 53.6 years (95% CI 51.6–55.5, *I* ^2^ = 95.5%) compared with the studies that did not select participants based on T2DM status (47.0 years, 95% CI 43.9–50.1, *I* ^2^ = 99.8%). The mean age of participants with a confirmed diagnosis of MASLD was not significantly different from those without (47.9 years [95% CI 45.4–50.4, *I* ^2^ = 99.7%] and 50.7 years [95% CI 47.6–53.9, *I* ^2^ = 99.3%], respectively). Forty‐three studies reported on the sex ratio of their population (*n* = 30 538), with 16 063 men and 14 475 women (53% vs. 47%) overall. The mean BMI in the T2DM subgroup of studies at 27.5 kg/m^2^ (95% CI 26.1–28.9 kg/m^2^, *I* ^2^ = 97.8%) was significantly higher than in the remaining studies that did not select participants based on T2DM status: 24.8 kg/m^2^ (95% CI 23.7–25.8 kg/m^2^, *I* ^2^ = 99.4%).

### 3.4 Prevalence of MASLD in Participants From South Asia

**FIGURE 2:**
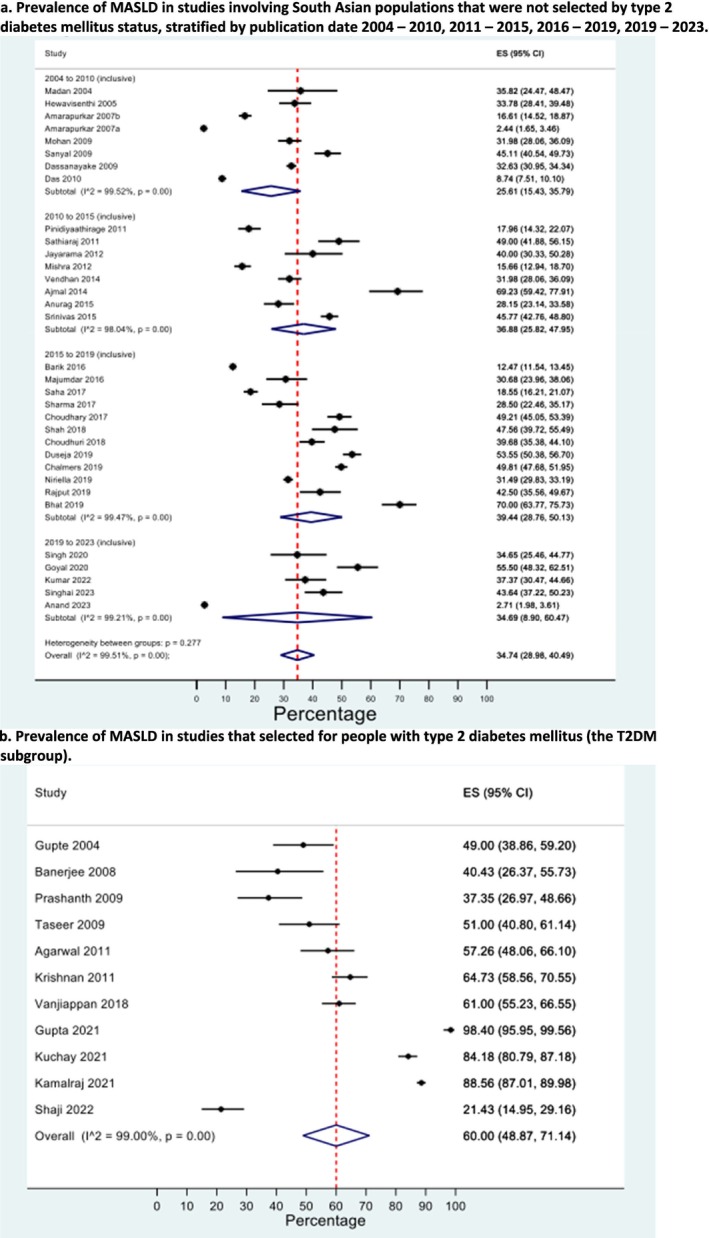
(a) Prevalence of MASLD in studies involving general South Asian populations that were not selected by Type 2 diabetes mellitus status, stratified by publication date 2004–2010, 2011–2015, 2016–2019, and 2019–2023. Key: CI: confidence interval, *I* ^2^: inconsistency index, MASLD: metabolic dysfunction–associated steatotic liver disease. (b) Prevalence of MASLD in studies that selected for people with Type 2 diabetes mellitus (the T2DM subgroup). Key: CI: confidence interval, *I* ^2^: inconsistency index, MASLD: metabolic dysfunction–associated steatotic liver disease, T2DM: Type 2 diabetes mellitus.

The pooled prevalence of MASLD in studies that did not select participants based on T2DM status was 34.7% (95% CI 29.0%–40.5%, *I* ^2^ = 99.5%) (Figure 2a). There was no significant difference in the estimation of prevalence based on publication date, although direct statistical comparison was not possible. In the T2DM subgroup, the pooled prevalence of MASLD was significantly higher at 60.0% (95% CI 48.9%–71.1%, *I* ^2^ = 99.0%) (Figure 2b). However, both calculated pooled prevalence results showed strong evidence of funnel plot asymmetry using Egger's test, with *p* = 0.0002 for the prevalence in studies not selecting for T2DM status and *p* < 0.0001 for the T2DM subgroup (Figures S2a and S2b).

Estimates of prevalence were consistent regardless of the method of MASLD diagnosis. The pooled prevalence in studies that did not select participants based on T2DM status, where diagnosis was via ultrasound, was 35.7% (95% CI 29.5%–41.9%, *I* ^2^ = 99.25%) (Figure S3a) and was comparable with 36.9% (95% CI 16.7%–57.6%, *I* ^2^ = 99.2%) in studies where MASLD was diagnosed by biopsy (Figure S3b).

**FIGURE 3:**
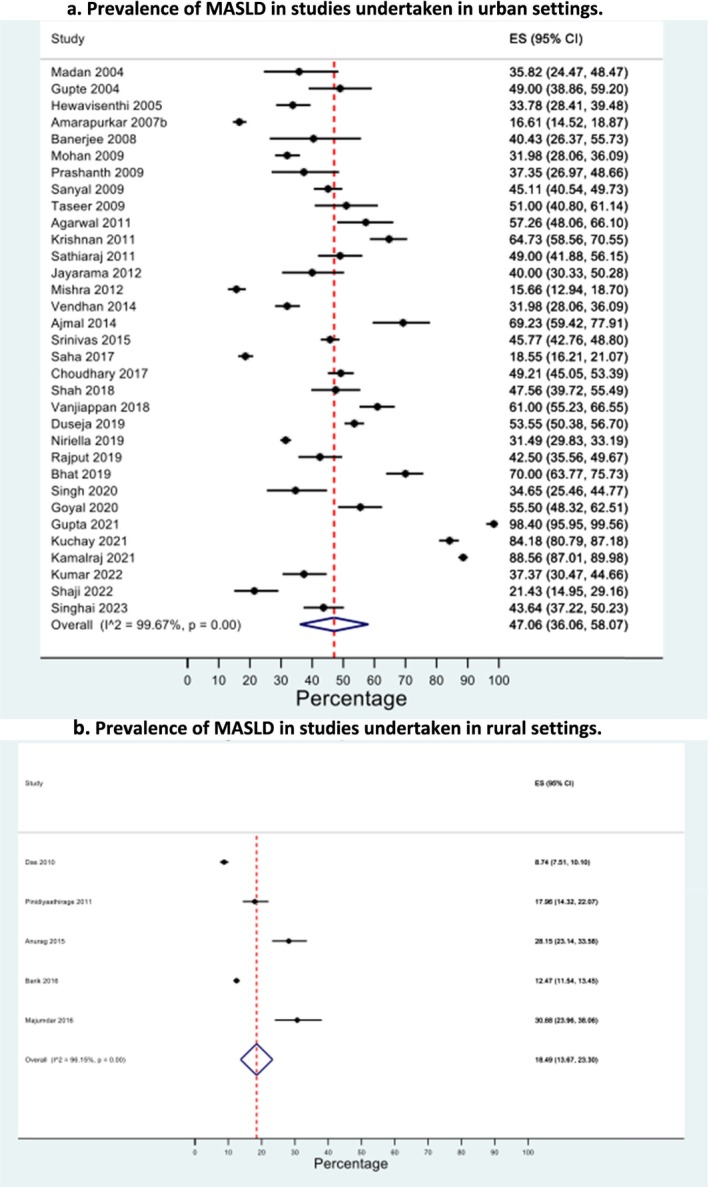
(a) Prevalence of MASLD in studies undertaken in urban settings. Key: CI: confidence interval, *I* ^2^: inconsistency index, MASLD: metabolic dysfunction–associated steatotic liver disease. (b) Prevalence of MASLD in studies undertaken in rural settings. Key: CI: confidence interval, *I* ^2^: inconsistency index, MASLD: metabolic dysfunction–associated steatotic liver disease.

The majority of studies that contained data on urban or rural settings were in cities (33 studies) compared with only five studies reporting on a rural population. Nevertheless, the pooled prevalence of MASLD in the urban population was significantly higher at 47.1% (95% CI 36.1%–58.1%, *I* ^2^ = 99.7%) (Figure 3a) compared with 18.5% (95% CI 13.7%–23.3%, *I* ^2^ = 96.2%) in rural participants (Figure 3b). There was good evidence of funnel plot symmetry for the urban prevalence meta‐analysis, with an Eggers *p*‐value of 0.54, suggesting an unlikely chance of publication bias; however, there was a high likelihood of publication bias for the rural prevalence meta‐analysis (Eggers result *p* < 0.0001), with associated funnel plot asymmetry (Figures S4a and S4b). Community‐based studies reported a lower prevalence than secondary care‐based studies: 25.4% (95% CI 17.9%–32.97%, *I* ^2^ = 99.6%), compared with 46.8% (95% CI 31.5%–62.1%, *I* ^2^ = 99.8%), respectively, albeit with overlapping CIs (Figures S5a and S5b).

Fifty‐three of the included studies were based in India, of which 37 reported on prevalence, with a calculated pooled prevalence of 42.5% (95% CI 31.7%–53.3%, *I* ^2^ = 99.9%). Of these studies, 44 reported on a primarily urban population, with 28 using ultrasound as the sole method of diagnosis. Seven studies based in populations outside of India had a pooled prevalence of 32.2% (95% CI 26.1%–38.3%, *I* ^2^ = 96.6%), primarily from participants from an urban population, diagnosed with MASLD primarily via ultrasound.

### 3.5 Severity of MASLD in Participants From South Asia

Alanine aminotransferase (ALT) was recorded in 16 out of 39 studies, with aspartate aminotransferase (AST) recorded in 15 studies. The calculated average mean ALT in MASLD was 49.4 IU (95% CI 34.1–64.8 IU, *I* ^2^ = 99.9%). In the T2DM subgroup of studies, the average mean ALT was 36.1 IU (95% CI 30.2–42.9 IU, *I* ^2^ = 96.0%). The average mean AST in MASLD in all studies was 36.4 IU (95% CI 26.2–46.5 IU, *I* ^2^ = 99.9%), with an average mean of 34.4 IU (95% CI 29.8–39.1 IU, *I* ^2^ = 97.0%) in the T2DM subgroup of studies.

Seventeen studies (*n* = 7613 participants) reported the severity of ultrasound‐determined steatosis (S1–S3), 12 in studies that did not select participants based on T2DM status and five in the T2DM subgroup. The calculated pooled proportional percentages in studies that did not select based on T2DM status for S1, S2, and S3 steatosis were 53.8% (95% CI 38.9%–68.7%, *I* ^2^ = 98.3%), 32.7% (95% CI 24.3%–41.2%, *I* ^2^ = 95.1%), and 15.4% (95% CI 7.7%–23.2%, *I* ^2^ = 97.9%), respectively (Figure S6a–c). In the T2DM subgroup of studies, the pooled proportional percentages for S1, S2, and S3 steatosis were 42.5% (95% CI 12.5%–72.5%, *I* ^2^ = 99.7%), 33.4% (95% CI 20.1%–46.9%, *I* ^2^ = 97.8%), and 23.8% (95% CI 2.0%–45.5%, *I* ^2^ = 99.8%), respectively (Figure S7a–c). Sensitivity analysis found that Gupta et al. [36] had a significant influence on the steatosis grade of the other studies (85% S3 in a tertiary center with the highest BMI in the study population). The proportion of S3 steatosis dropped from 23.8% to 7.0% (95% CI 2.2%–11.8%, *I* ^2^ = 93.4%) in the T2DM subgroup of studies when the study was removed (Figure S7d).

**FIGURE 4:**
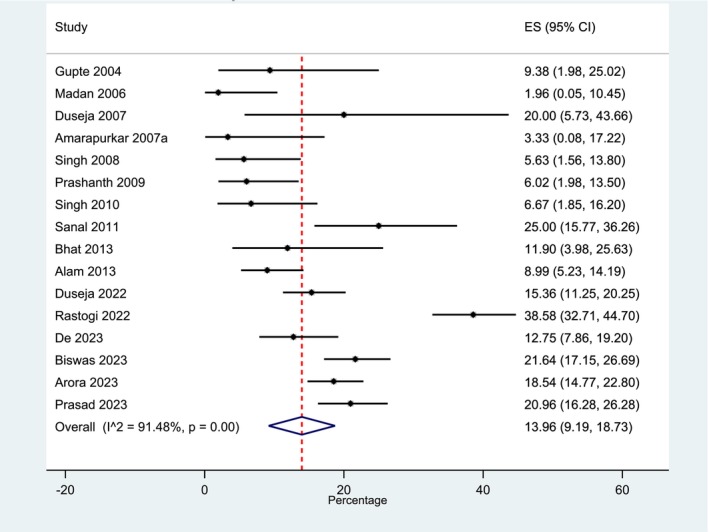
Pooled proportional percentage of participants with advanced fibrosis (NAS fibrosis stage ≥ F3) in all studies. Key: NAS: nonalcoholic fatty liver disease activity score.

Only 16 studies reported the histological assessment of fibrosis (15 in India and 1in Bangladesh; total participants *n* = 9431). No meta‐analysis was possible for the T2DM subgroup (*n* = 183 participants) as only two studies contained information on the breakdown of fibrosis stage [37, 38]. Overall, the pooled average proportional percentage of participants with F0 fibrosis was 35.8% (95% CI 28.1%–43.5%, *I* ^2^ = 94.2%); F1 fibrosis, 33.8% (95% CI 27.9%–39.8%, *I* ^2^ = 89.2%); and F2 fibrosis, 14.6% (95% CI 9.4–19.8, *I* ^2^ = 96.7%) (Figure S8a–c). The pooled average proportion of participants with advanced fibrosis (≥ F3) was 14.0% (95% CI 9.2%–18.7%, *I* ^2^ = 91.5%) (Figure 4).

### 3.6 Clinical Outcomes for Participants From South Asia With MASLD

Four studies reported on clinical outcomes of interest: hepatic decompensation (encompassing variceal bleeding, encephalopathy, ascites, and jaundice), development of HCC, and death [32, 33, 34, 35]. However, three had different endpoints, with only one study reporting on all clinical outcomes of interest [35], so it was not possible to combine the data into a meta‐analysis.

### 3.7 Results of Quality Assessment of Included Studies

A breakdown of scores using the NIH Quality Assessment Tools [21] is outlined in Table S2. Three studies had a score of < 5, 31 studies had a score of 5–7, and 28 studies had a score of > 7 (highest score 10). The majority of studies were cross‐sectional and, as such, were limited by their design, preventing measurement of exposure and outcome at separate time intervals. We performed a subanalysis of higher quality studies (score > 7), of which there were 28. There was no significant change in prevalence or severity distribution of MASLD in this population, nor in the *I* ^2^ inconsistency index values for the meta‐analyses.

## 4 Discussion

The increasing burden of MASLD both in primary and secondary care is a major challenge at the forefront of hepatology. Given its rapidly increasing prevalence, health services worldwide need effective strategies to manage patients safely and efficiently. Patients of South Asian ethnicities represent a large proportion of the global population, yet little is known about MASLD epidemiology, severity, or outcomes in this group. Our systematic review with meta‐analysis has provided data on the pooled prevalence of MASLD in patients of South Asian ethnicities, both in study populations unselected for T2DM status and in studies of participants with T2DM. To the best of our knowledge, we are the first to calculate pooled proportional percentages of cross‐sectional severity of steatosis grade on ultrasound and fibrosis on biopsy in this cohort. We have highlighted a large variation between the included studies, the majority of which have been cross‐sectional in design, along with a relative paucity of data on longitudinal patient outcomes for liver decompensation, development of HCC, and death in South Asian populations.

We estimate the overall prevalence of MASLD to be 34.7% in the South Asian population from both primary and secondary care in the four countries represented in this dataset. This is similar to a previous estimate in the South Asian population at 33.8% by Younossi and colleagues [39] and higher than the prevalence of MASLD in all other global regions apart from Latin America [39]. The estimated prevalence of 60% in studies that included only participants with T2DM was similar to that calculated in a previous meta‐analysis (60.2% in Pakistan and 67.7% in India [8]) and is in keeping with known risks associated with the metabolic syndrome [40]. Patients in the T2DM subgroup of studies were older than patients in studies where investigators did not select based on T2DM status (53.6 years vs. 47.0 years).

We have also distinguished MASLD prevalence between urban and rural populations in the South Asian population, with a greater prevalence in the urban population (47.1% vs. 18.5%). This could be explained by the higher metabolic risk factor burden in the urban South Asian population [41] and serves to highlight that MASLD remains a disease influenced by socioeconomic and cultural factors. This has implications on the delivery of future care pathways, where health interventions in urban populations may require a different approach to those in rural settings.

We also found a difference in the prevalence of MASLD from studies based in the community versus secondary care: 25.4% and 46.8%, respectively. However, it is unclear whether this demonstrates preselection bias based on effective risk stratification of disease and referral to specialist hepatology services. Only six studies reported the use of noninvasive tests such as VCTE and FIB4; however, these were not used in the context of stratifying referrals to secondary care or in conjunction with biopsy to confirm fibrosis stage. Low utilization of noninvasive tests generally may, in part, be explained by limited access and the perceived need for specialist interpretation of diagnostic thresholds, hindering use in resource‐poor community settings [42].

In histology‐based studies, 14.0% of participants had advanced fibrosis (≥ F3). Previous studies in multiethnic cohorts in the United Kingdom and the United States reported prevalence of 7.7% and 5.6%, respectively [43, 44], both using noninvasive test risk stratification: FIB4 and ELF testing and Magnetic resonance imaging proton density fat fraction (MRI‐PDFF) and biopsy, respectively. It is unclear if the selection of more severe, metabolically high‐risk patients for biopsy in the studies included in our meta‐analysis affected the prevalence estimate of those with ≥ F3 fibrosis, and cautious interpretation of comparisons between our pooled proportion value and the other aforementioned multiethnic cohort studies is necessary. Further research with high‐quality, longitudinal studies into the development and implementation of noninvasive tests in populations of South Asian ethnicities is needed. This will feed forward into improved risk stratification pathways to select patients who will benefit from liver biopsy, leading to more effective care.

Our meta‐analysis was limited by several factors. First, we did not identify any studies that contained relevant data on South Asian ethnicities as a stand‐alone group alongside comparator ethnicities that reported on prevalence, severity, or clinical outcomes. As such, we were unable to conduct a head‐to‐head analysis or calculate odds ratios comparing South Asian ethnicities with another ethnicity. Given that this cohort represents the largest growing population of patients with MASLD globally [18], it was surprising that there was little published evidence as to whether this translated into poorer long‐term outcomes for patients of South Asian ethnicities directly compared to other ethnicities.

Another limitation was that the majority of published studies included were observational; therefore, a meta‐analysis of the incidence of MASLD in the South Asian general population was not possible. We also acknowledge the effect of publication bias on the overall pooled prevalence and breakdown of histological severity seen in the funnel plots we have created. Finally, we were unable to identify studies in our searches that compared people currently living in South Asian countries with participants of South Asian ethnicity who have migrated to other countries outside of South Asia.

In our analysis, we have chosen to focus on studies involving adult patients aged 18 or above with MASLD; however, we and others have reported a substantial burden of steatotic liver disease within the pediatric and adolescent population [45, 46]. Future meta‐analyses to delineate the epidemiology in this cohort would be of great benefit, adding to our knowledge of disease course and prevention.

Included studies were limited by substantial heterogeneity, with *I* ^2^ values exceeding 75%. Inclusion criteria were generally consistent, albeit with variation in age cut‐offs; however, study populations differed widely in their setting and in their implementation of diagnostic tests for MASLD. Attempts to counter bias in the interpretation of results within studies also varied, with some studies blinding assessors to patient demographic and clinical information, whereas others did not. Several studies also extracted data from patient notes and databases, with inherent limitations associated with routine care records such as missing data points and incomplete coding. Our subanalysis of higher quality studies also failed to impact on the high *I* ^2^ values in our meta‐analyses. Taken together, heterogeneity and study design are intrinsic limitations of the literature, and we are unable to make strong inferences regarding the wider South Asian population as a whole, but this has identified future opportunities for further work.

South Asian ethnicities encompass participants from a large geographic area, including a quarter of the world's population with varied cultural heritages, dietary habits, social structures, health beliefs, and healthcare systems [47]. Ethnicity acts as a compound surrogate for these and many other factors. Genetic heterogeneity can be as diverse within a reported ethnicity as between different ethnic groups, so classifying several populations under the umbrella of a single ethnicity is likely an oversimplification. Ethnicity and socioeconomic status are inextricably linked and have a huge impact on disparities noted in health outcomes, but adjusting for this can be challenging, especially when analyzing data retrospectively. Finally, both ethnicity and race are often self‐reported and, as such, can be unreliable labels under which to group patients. Despite these limitations, we believe there is merit in analyzing outcomes in ethnic groups in order to identify gaps in knowledge and areas for further improvement in the care of patients.

Thus, although patients of South Asian ethnicity represent a distinct phenotype in MASLD, the interpretation of why this is and which patients are most at risk remains a challenge.

## 5 Conclusion

We have reported pooled prevalence estimates of MASLD in people of South Asian ethnicities, showing higher rates in people with T2DM and those living in urban environments. We have found that the proportion of patients with advanced fibrosis may be higher than in other multiethnic populations, though more high‐quality longitudinal studies utilizing noninvasive testing in both the community and secondary care and assessing outcomes with regard to decompensation, HCC risk, and mortality are needed to help characterize and address the growing disease burden in South Asian populations.

## Conflicts of Interest

The authors declare no conflicts of interest.

## Supporting information


**Figure S1:** The electronic search terms in different databases.
**Figure S2a:** Funnel plot of studies estimating the percentage prevalence of MASLD in studies that did not select participants based on T2DM status (n = 33). Egger's result: p = 0.0002, which is highly significant. There is strong evidence of funnel plot asymmetry that suggests the presence of small‐study effects, which may indicate potential publication bias in a meta‐analysis.
**Figure S2b:** Funnel plot of studies estimating the percentage prevalence of MASLD in studies that selected for T2DM (the T2DM subgroup) (n = 11). Egger's result: p < 0.0001, which is highly significant. There is strong evidence of funnel plot asymmetry that suggests the presence of small‐study effects, which may indicate potential publication bias in a meta‐analysis.
**Figure S3a:** Prevalence of MASLD diagnosed by ultrasound in studies that did not select for patients by T2DM status.
**Figure S3b:** Prevalence of MASLD diagnosed by biopsy in studies that did not select for patients by T2DM status (MASLD: metabolic dysfunction–associated steatotic liver disease, T2DM: Type 2 diabetes mellitus, CI: confidence interval, I2: inconsistency index).
**Figure S4a:** Funnel plot of studies estimating the percentage prevalence of MASLD undertaken in urban settings. Egger's result: p = 0.5358, which is not significant. There is good evidence of funnel plot symmetry that suggests that publication bias is unlikely.
**Figure S4b:** Funnel plot of studies estimating the percentage prevalence of MASLD undertaken in rural settings. Egger's result: p < 0.0001, which is highly significant. There is strong evidence of funnel plot asymmetry that suggests the presence of small‐study effects, which may indicate potential publication bias in a meta‐analysis.
**Figure S5a:** Prevalence of MASLD in studies undertaken in the community.
**Figure S5b:** Prevalence of MASLD in studies undertaken in secondary care.
**Figure S6a:** Pooled proportional percentage of participants with S1 steatosis in studies that did not select for patients based on T2DM status.
**Figure S6b:** Pooled proportional percentage of participants with S2 steatosis in studies that did not select for patients based on T2DM status (T2DM: Type 2 diabetes mellitus, CI: confidence interval, I2: inconsistency index).
**Figure S6c:** Pooled proportional percentage of participants with S3 steatosis in studies that did not select for patients based on T2DM status (T2DM: Type 2 diabetes mellitus, CI: confidence interval, I2: inconsistency index).
**Figure S7a:** Pooled proportional percentage of S1 steatosis in the T2DM subgroup.
**Figure S7b:** Pooled proportion of S2 steatosis in the T2DM subgroup.
**Figure S7c:** Pooled proportion of S3 steatosis in the T2DM subgroup.
**Figure S8a:** Pooled proportional percentage of participants with NAS fibrosis stage F0 fibrosis in all studies.
**Figure S8b:** Pooled proportional percentage of participants with NAS fibrosis stage F1 fibrosis in all studies.
**Figure S8c:** Pooled proportional percentage of participants with NAS fibrosis stage F2 fibrosis in all studies.
**Table S1:** Breakdown of study level characteristics included in systematic review and meta‐analysis (N.B. study setting described as hospital: secondary care clinic/inpatient setting, community: primary care, rural and urban were defined based on individual study description).
**Table S2:** Breakdown of NIH (National Institute of Health) Quality Assessment Tool scores for included studies. Numerical columns correspond to questions in either relevant assessment tool: Observational cohort/cross‐sectional studies or case–control studies [16].

## Data Availability

The data that support the findings of this study are available from the corresponding author upon reasonable request.
