# Supplementary material for: Epidemiology of Metabolic Dysfunction–Associated Steatotic Liver Disease in South Asian Ethnicities: A Systematic Review and Meta‐Analysis
Source: J Gastroenterol Hepatol. 2025 Sep 26;40(12):2830–41. doi: 10.1111/jgh.70080 (PMC12666614; doi:10.1111/jgh.70080)
Supplement: Supplementary file 1 — Figure S1: The electronic search terms in different databases. Figure S2a: Funnel plot of studies estimating the percentage prevalence of MASLD in studies that did not select participants based on T2DM status (n = 33). Egger's result: p = 0.0002, which is highly significant. There is strong evidence of funnel plot asymmetry that suggests the presence of small‐study effects, which may indicate potential publication bias in a meta‐analysis. Figure S2b: Funnel plot of studies estimating the percentage prevalence of MASLD in studies that selected for T2DM (the T2DM subgroup) (n = 11). Egger's result: p < 0.0001, which is highly significant. There is strong evidence of funnel plot asymmetry that suggests the presence of small‐study effects, which may indicate potential publication bias in a meta‐analysis. Figure S3a: Prevalence of MASLD diagnosed by ultrasound in studies that did not select for patients by T2DM status. Figure S3b: Prevalence of MASLD diagnosed by biopsy in studies that did not select for patients by T2DM status (MASLD: metabolic dysfunction–associated steatotic liver disease, T2DM: Type 2 diabetes mellitus, CI: confidence interval, I2: inconsistency index). Figure S4a: Funnel plot of studies estimating the percentage prevalence of MASLD undertaken in urban settings. Egger's result: p = 0.5358, which is not significant. There is good evidence of funnel plot symmetry that suggests that publication bias is unlikely. Figure S4b: Funnel plot of studies estimating the percentage prevalence of MASLD undertaken in rural settings. Egger's result: p < 0.0001, which is highly significant. There is strong evidence of funnel plot asymmetry that suggests the presence of small‐study effects, which may indicate potential publication bias in a meta‐analysis. Figure S5a: Prevalence of MASLD in studies undertaken in the community. Figure S5b: Prevalence of MASLD in studies undertaken in secondary care. Figure S6a: Pooled proportional percentage of participants with S1 stea [file JGH-40-2830-s001.docx]

**Supplementary Tables and Figures**

**List of supplementary materials:**

- **Supplementary Figure 1. The electronic search terms in different databases.**
- **Supplementary Figure 2a-b. Funnel plot of studies estimating percentage prevalence of MASLD in studies that did not select participants based on T2DM status and the T2DM subgroup of studies.**
- **Supplementary Figure 3a-c. Prevalence of MASLD diagnosed by ultrasound and biopsy in studies that did not select for patients by T2DM status.**
- **Supplementary Figure 4a-b. Funnel plot of studies estimating percentage prevalence of MASLD undertaken in urban and rural settings.**
- **Supplementary Figure 5a-b. Prevalence of MASLD in studies undertaken in the community and in secondary care**
- **Supplementary Figure 6a-c. Pooled proportional percentage of participants with S1-3 steatosis in studies that did not select for patients based on T2DM status.**
- **Supplementary Figure 7a-d. Pooled proportional percentage of S1-3 steatosis in the T2DM subgroup.**
- **Supplementary Figure 8a-c. Pooled proportional percentage of participants with NAS Fibrosis stage F0-2 fibrosis in all studies.**
- **Supplementary Table 1.** Breakdown of studies included in systematic review and meta-analysis.
- **Supplementary Table 2.** Breakdown of NIH Quality Assessment Tool scores for included studies.

**Supplementary Figure 1. The electronic search terms in different databases.**

| **Database** | **No. of results** |
| --- | --- |
| PubMed | 873 |
| Embase | 2167 |
| Cochrane | 53 |
| Ovid Medline | 93 |

**Pubmed search terms: 864 articles**

**((((((((NAFLD) OR (non alcoholic fatty liver disease)) OR (Fatty liver)) OR (NASH)) OR (non alcoholic steatohepatitis)) OR (steatohepatitis)) AND ((((((prevalence) OR (severity)) OR (epidemiology)) OR (epidemiol*)) OR (risk score)) OR (score))) AND ((((((complications) OR (decompensat*)) OR (cirrhosis)) OR (hepatocellular carcinoma)) OR (HCC)) OR (death))) AND ((((((south asian) OR (south asia*)) OR (india*)) OR (pakistan*)) OR (sri lanka*)) OR (bangladesh*))**

**Embase search terms: 2166 articles**

1. nafld OR 'non alcoholic fatty liver disease'/exp OR 'non alcoholic fatty liver disease' OR (non AND ('alcoholic'/exp OR alcoholic) AND fatty AND ('liver'/exp OR liver) AND ('disease'/exp OR disease)) OR 'fatty liver'/exp OR 'fatty liver' OR (fatty AND ('liver'/exp OR liver)) OR nash OR 'non alcoholic steatohepatitis'/exp OR 'non alcoholic steatohepatitis' OR (non AND ('alcoholic'/exp OR alcoholic) AND ('steatohepatitis'/exp OR steatohepatitis)) OR 'steatohepatitis'/exp OR steatohepatitis
2. 'prevalence'/exp OR prevalence OR 'severity'/exp OR severity OR 'epidemiology'/exp OR epidemiology OR epidemiol* OR 'risk score'/exp OR 'risk score' OR (('risk'/exp OR risk) AND ('score'/exp OR score)) OR 'score'/exp OR score
3. 'complications'/exp OR complications OR decompensat* OR 'cirrhosis'/exp OR cirrhosis OR 'hepatocellular carcinoma'/exp OR 'hepatocellular carcinoma' OR (hepatocellular AND ('carcinoma'/exp OR carcinoma)) OR hcc OR 'death'/exp OR death
4. 'south asian'/exp OR 'south asian' OR (south AND ('asian'/exp OR asian)) OR (south AND asia*) OR india* OR pakistan* OR (sri AND lanka*) OR bangladesh*

**Cochrane search terms: 52 articles**

#1 (NAFLD):ti,ab,kw (Word variations have been searched)

#2 non alcoholic fatty liver disease

#3 NASH

#4 non alcoholic steatohepatitis

#5 steatohepatitis

#6 #1 or #2 or #3 or #4 or #5

#7 prevalence

#8 severity

#9 epidemiol*

#10 risk

#11 score

#12 #7 or #8 or #9 or #10 or #11

#13 complications

#14 decompensat*

#15 cirrhosis

#16 hepatocellular carcinoma

#17 HCC

#18 death

#19 #13 or #14 or #15 or #16 or #17 or #18

#20 "south asian"

#21 "south asia*"

#22 india*

#23 pakistan*

#24 sri lanka*

#25 bangladesh*

#26 #20 or #21 or #22 or #23 or #24 or #25

#27 #6 and #12 and #19 and #26

**Ovid Medline search terms: 93 articles**

1. NAFLD.mp. [mp=title, book title, abstract, original title, name of substance word, subject heading word, floating sub-heading word, keyword heading word, organism supplementary concept word, protocol supplementary concept word, rare disease supplementary concept word, unique identifier, synonyms] 21860

2. non alcoholic fatty liver disease.mp. [mp=title, book title, abstract, original title, name of substance word, subject heading word, floating sub-heading word, keyword heading word, organism supplementary concept word, protocol supplementary concept word, rare disease supplementary concept word, unique identifier, synonyms] 26786

3. NASH.mp. [mp=title, book title, abstract, original title, name of substance word, subject heading word, floating sub-heading word, keyword heading word, organism supplementary concept word, protocol supplementary concept word, rare disease supplementary concept word, unique identifier, synonyms] 12359

4. Non alcoholic steatohepatitis.mp. [mp=title, book title, abstract, original title, name of substance word, subject heading word, floating sub-heading word, keyword heading word, organism supplementary concept word, protocol supplementary concept word, rare disease supplementary concept word, unique identifier, synonyms] 5736

5. steatohepatitis.mp. [mp=title, book title, abstract, original title, name of substance word, subject heading word, floating sub-heading word, keyword heading word, organism supplementary concept word, protocol supplementary concept word, rare disease supplementary concept word, unique identifier, synonyms] 15594

6. Non-alcoholic Fatty Liver Disease/ 20565

7. 1 or 2 or 3 or 4 or 5 or 6 38983

8. prevalence.mp. [mp=title, book title, abstract, original title, name of substance word, subject heading word, floating sub-heading word, keyword heading word, organism supplementary concept word, protocol supplementary concept word, rare disease supplementary concept word, unique identifier, synonyms] 839310

9. Prevalence/ 335346

10. epidemiol*.mp. [mp=title, book title, abstract, original title, name of substance word, subject heading word, floating sub-heading word, keyword heading word, organism supplementary concept word, protocol supplementary concept word, rare disease supplementary concept word, unique identifier, synonyms] 2282471

11. 8 or 9 or 10 2647021

12. severity.mp. [mp=title, book title, abstract, original title, name of substance word, subject heading word, floating sub-heading word, keyword heading word, organism supplementary concept word, protocol supplementary concept word, rare disease supplementary concept word, unique identifier, synonyms] 724804

13. risk.mp. [mp=title, book title, abstract, original title, name of substance word, subject heading word, floating sub-heading word, keyword heading word, organism supplementary concept word, protocol supplementary concept word, rare disease supplementary concept word, unique identifier, synonyms] 3047171

14. 12 or 13 3582836

15. complications.mp. [mp=title, book title, abstract, original title, name of substance word, subject heading word, floating sub-heading word, keyword heading word, organism supplementary concept word, protocol supplementary concept word, rare disease supplementary concept word, unique identifier, synonyms] 3264843

16. decompensat*.mp. [mp=title, book title, abstract, original title, name of substance word, subject heading word, floating sub-heading word, keyword heading word, organism supplementary concept word, protocol supplementary concept word, rare disease supplementary concept word, unique identifier, synonyms] 25843

17. cirrhosis.mp. [mp=title, book title, abstract, original title, name of substance word, subject heading word, floating sub-heading word, keyword heading word, organism supplementary concept word, protocol supplementary concept word, rare disease supplementary concept word, unique identifier, synonyms] 145103

18. hepatocellular carcinoma.mp. [mp=title, book title, abstract, original title, name of substance word, subject heading word, floating sub-heading word, keyword heading word, organism supplementary concept word, protocol supplementary concept word, rare disease supplementary concept word, unique identifier, synonyms] 108694

19. HCC.mp. [mp=title, book title, abstract, original title, name of substance word, subject heading word, floating sub-heading word, keyword heading word, organism supplementary concept word, protocol supplementary concept word, rare disease supplementary concept word, unique identifier, synonyms] 71128

20. death.mp. [mp=title, book title, abstract, original title, name of substance word, subject heading word, floating sub-heading word, keyword heading word, organism supplementary concept word, protocol supplementary concept word, rare disease supplementary concept word, unique identifier, synonyms] 932220

21. 15 or 16 or 17 or 18 or 19 or 20 4206963

22. "south asian".mp. [mp=title, book title, abstract, original title, name of substance word, subject heading word, floating sub-heading word, keyword heading word, organism supplementary concept word, protocol supplementary concept word, rare disease supplementary concept word, unique identifier, synonyms] 6615

23. "south asia*".mp. [mp=title, book title, abstract, original title, name of substance word, subject heading word, floating sub-heading word, keyword heading word, organism supplementary concept word, protocol supplementary concept word, rare disease supplementary concept word, unique identifier, synonyms] 11828

24. india*.mp. [mp=title, book title, abstract, original title, name of substance word, subject heading word, floating sub-heading word, keyword heading word, organism supplementary concept word, protocol supplementary concept word, rare disease supplementary concept word, unique identifier, synonyms] 253503

25. pakistan*.mp. [mp=title, book title, abstract, original title, name of substance word, subject heading word, floating sub-heading word, keyword heading word, organism supplementary concept word, protocol supplementary concept word, rare disease supplementary concept word, unique identifier, synonyms] 33997

26. sri lanka*.mp. [mp=title, book title, abstract, original title, name of substance word, subject heading word, floating sub-heading word, keyword heading word, organism supplementary concept word, protocol supplementary concept word, rare disease supplementary concept word, unique identifier, synonyms] 10516

27. bangladesh*.mp. [mp=title, book title, abstract, original title, name of substance word, subject heading word, floating sub-heading word, keyword heading word, organism supplementary concept word, protocol supplementary concept word, rare disease supplementary concept word, unique identifier, synonyms] 21024

28. 22 or 23 or 24 or 25 or 26 or 27 316448

29. 7 and 11 and 14 and 21 and 28 94

**Supplementary Figure 2a. Funnel plot of studies estimating percentage prevalence of MASLD in studies that did not select participants based on T2DM status (n = 33). Egger’s Result: p=0.0002, which is highly significant. There is strong evidence of funnel plot asymmetry which suggests the presence of small-study effects, which may indicate potential publication bias in a meta-analysis.**


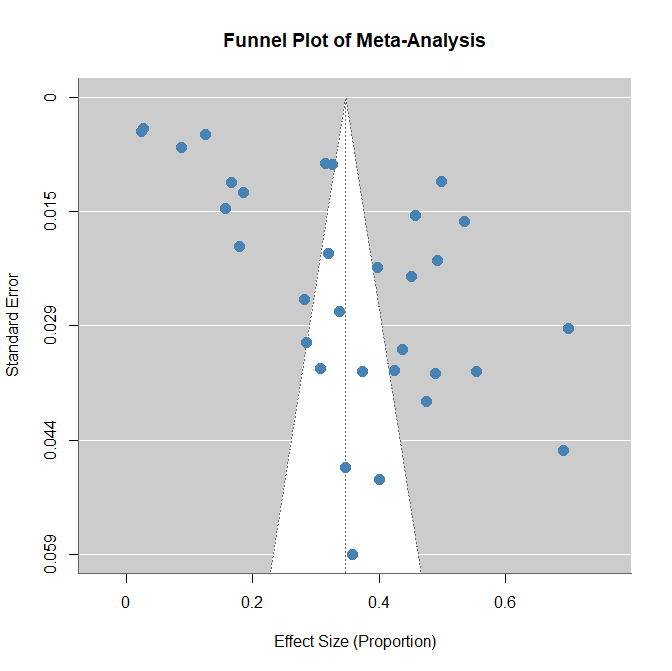


**Key: MASLD: Metabolic dysfunction associated steatotic liver disease, T2DM: Type 2 Diabetes Mellitus.**

**Supplementary Figure 2b. Funnel plot of studies estimating percentage prevalence of MASLD in studies that selected for T2DM (the T2DM subgroup) (n = 11). Egger’s Result: p<0.0001, which is highly significant. There is strong evidence of funnel plot asymmetry which suggests the presence of small-study effects, which may indicate potential publication bias in a meta-analysis.**


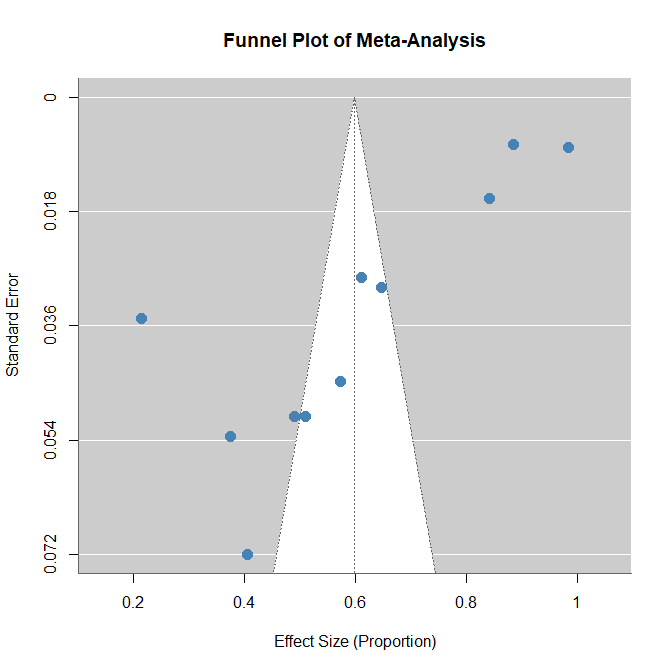


**Key: MASLD: Metabolic dysfunction associated steatotic liver disease, T2DM: Type 2 Diabetes Mellitus.**

**Supplementary Figure 3a. Prevalence of MASLD diagnosed by ultrasound in studies that did not select for patients by T2DM status.**
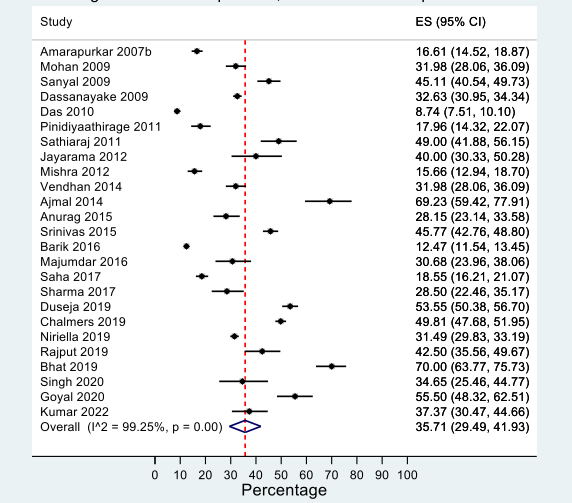


**Key: MASLD: Metabolic dysfunction associated steatotic liver disease, T2DM: Type 2 Diabetes Mellitus, CI: confidence interval, I^2^: inconsistency index.**

**Supplementary Figure 3b. Prevalence of MASLD diagnosed by biopsy in studies that did not select for patients by T2DM status (MASLD: Metabolic dysfunction associated steatotic liver disease, T2DM: Type 2 Diabetes Mellitus, CI: confidence interval, I^2^: inconsistency index).
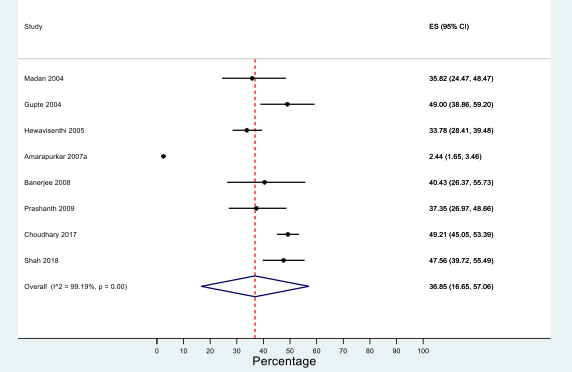
**

**Supplementary Figure 4a. Funnel plot of studies estimating percentage prevalence of MASLD undertaken in urban settings. Egger’s Result: p=0.5358, which is not significant. There is good evidence of funnel plot symmetry which suggests that publication bias is unlikely.**


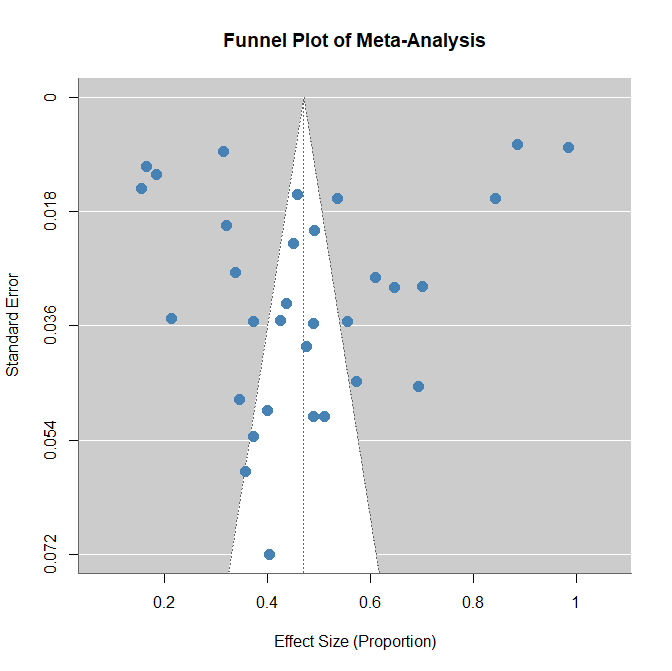


**Key: MASLD: Metabolic dysfunction associated steatotic liver disease.**

**Supplementary Figure 4b. Funnel plot of studies estimating percentage prevalence of MASLD undertaken in rural settings. Egger’s Result: p<0.0001, which is highly significant. There is strong evidence of funnel plot asymmetry which suggests the presence of small-study effects, which may indicate potential publication bias in a meta-analysis.**


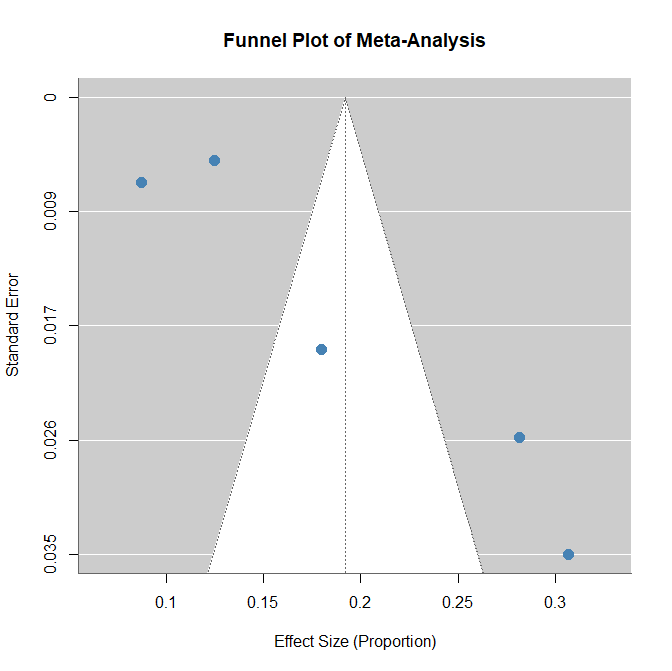


**Key: MASLD: Metabolic dysfunction associated steatotic liver disease.**

**Supplementary Figure 5a. Prevalence of MASLD in studies undertaken in the community.**

**
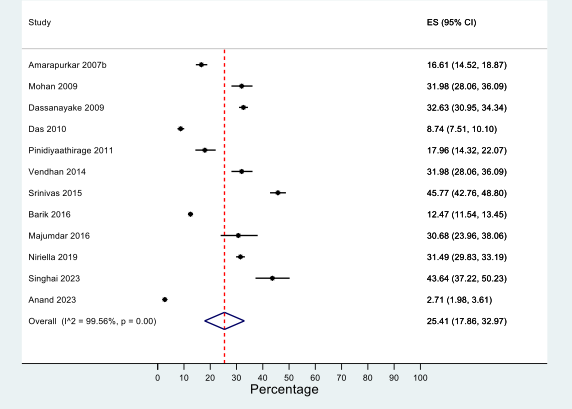
**

**Key: MASLD: Metabolic dysfunction associated steatotic liver disease, CI: confidence interval, I^2^: inconsistency index.**

**Supplementary Figure 5b. Prevalence of MASLD in studies undertaken in secondary care.**

**
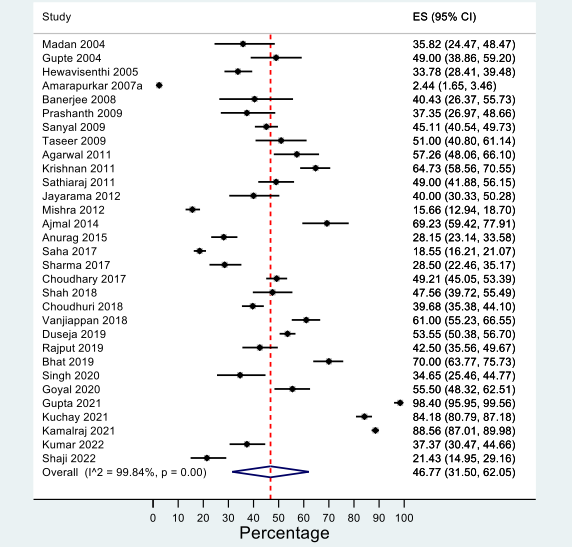
**

**Key: MASLD: Metabolic dysfunction associated steatotic liver disease, CI: confidence interval, I^2^: inconsistency index.**

**Supplementary Figure 6a. Pooled proportional percentage of participants with S1 steatosis in studies that did not select for patients based on T2DM status.**

**Key: T2DM: Type 2 diabetes mellitus, CI: confidence interval, I^2^: inconsistency index.**

**Supplementary Figure 6b. Pooled proportional percentage of participants with S2 steatosis in studies that did not select for patients based on T2DM status (T2DM: Type 2 diabetes mellitus, CI: confidence interval, I^2^: inconsistency index).**

**Key: T2DM: Type 2 diabetes mellitus, CI: confidence interval, I^2^: inconsistency index.**

**Supplementary Figure 6c. Pooled proportional percentage of participants with S3 steatosis in studies that did not select for patients based on T2DM status (T2DM: Type 2 diabetes mellitus, CI: confidence interval, I^2^: inconsistency index).**

**Key: T2DM: Type 2 diabetes mellitus, CI: confidence interval, I^2^: inconsistency index.**

**Supplementary Figure 7a. Pooled proportional percentage of S1 steatosis in the T2DM subgroup.**

**Key: T2DM: Type 2 diabetes mellitus, CI: confidence interval, I^2^: inconsistency index.**

**Supplementary Figure 7b. Pooled proportion of S2 steatosis in the T2DM subgroup.**

**Key: T2DM: Type 2 diabetes mellitus, CI: confidence interval, I^2^: inconsistency index.**

**Supplementary Figure 7c. Pooled proportion of S3 steatosis in the T2DM subgroup.**

**Key: T2DM: Type 2 diabetes mellitus, CI: confidence interval, I^2^: inconsistency index.**

**Supplementary Figure 7d. Pooled proportion of S3 steatosis in the the T2DM subgroup with Gupta et al^[31]^ removed.**

**Key: T2DM: Type 2 diabetes mellitus, CI: confidence interval, I^2^: inconsistency index.**

**Supplementary Figure 8a. Pooled proportional percentage of participants with NAS Fibrosis stage F0 fibrosis in all studies.**

**Key: NAS: Non-Alcoholic Fatty Liver Disease Activity Score, CI: confidence interval, I^2^: inconsistency index.**

**Supplementary Figure 8b. Pooled proportional percentage of participants with NAS Fibrosis stage F1 fibrosis in all studies.**

**Key: NAS: Non-Alcoholic Fatty Liver Disease Activity Score, CI: confidence interval, I^2^: inconsistency index.**

**Supplementary Figure 8c. Pooled proportional percentage of participants with NAS Fibrosis stage F2 fibrosis in all studies.**

**Key: NAS: Non-Alcoholic Fatty Liver Disease Activity Score, CI: confidence interval, I^2^: inconsistency index.**

**Supplementary Table 1. Breakdown of study level characteristics included in systematic review and meta-analysis (N.B. study setting described as hospital: secondary care clinic/inpatient setting, community: primary care, rural and urban were defined based on individual study description).**

| **Author** | **Year** | **Journal** | **Study design** | **Data on Prevalance** | **Data on Severity** | **Data on MASLD endpoints** | **Total number of participants** | **Country** | **Study setting** | **Rural vs Urban setting** | **Diagnostic method for MASLD** |
| --- | --- | --- | --- | --- | --- | --- | --- | --- | --- | --- | --- |
| Amarapurkar et al. (2007) (a) | 2007 | Annals of Hepatology | Cross-sectional | Y | Y | N | 1230 | India | Hospital | Not documented | Biopsy (autopsy) |
| Amarapurkar et al. (2007) (b) | 2007 | Annals of Hepatology | Cross-sectional | Y | Y | N | 1168 | India | Community | Urban | US |
| Agarwal et al. | 2011 | Journal of the Association of the Physicians of India | Cross-sectional | Y | Y | N | 124 | India | Hospital | Urban | US |
| Ajmal et al. | 2014 | Indian Heart Journal | Cross-sectional | Y | Y | N | 104 | India | Hospital | Urban | US |
| Alam et al. | 2013 | World Journal of Hepatology | Cross-sectional | N | Y | N | 177 | Bangladesh | Hospital | Urban | US/Biopsy |
| Amarapurkar et al. | 2013 | Tropical Gastroenterology | Prospective cohort | N | N | Y (HCC) | 145 | India | Hospital | Urban | US/Biopsy |
| Anand et al. | 2023 | Journal of Clinical and Experimental Hepatology | Cross-sectional | Y | N | N | 1660 | India | Community | Urban/Rural population | FAST score (VCTE and AST) |
| Anurag et al. | 2015 | Tropical Gastroenterology | Cross-sectional | Y | Y | N | 302 | India | Hospital | Rural | US |
| Arora et al. | 2022 | European Journal of Clinical Nutrition | Randomised control trial | N | Y | N | 59 | India | Hospital | Urban | US |
| Arora et al. | 2023 | Digestive Diseases and Sciences | Cross-sectional | N | Y | x | 383 | India | Hospital | Urban | Biopsy |
| Banerjee et al. | 2008 | Journal of the Association of the Physicians of India | Cross-sectional | Y | Y | N | 47 | India | Hospital | Urban | US/Biopsy |
| Baqai et al. | 2023 | Journal of Ayub Medical College Abbottabad | Cross-sectional | N | Y | N | 255 | Pakistan | Hospital | Urban | US |
| Barik et al. | 2016 | International Journal of Cardiology | Cross-sectional | Y | N | N | 4691 | India | Community | Rural | US |
| Bhat et al. | 2013 | Tropical Gastroenterology | Cross-sectional | N | Y | N | 42 | India | Hospital | Urban | Biopsy |
| Bhat et al. | 2019 | Sleep and Breathing | Cross-sectional | Y | Y | N | 240 | India | Hospital | Urban | US |
| Biswas et al. | 2023 | Postgraduate Medical Journal | Retrospective analysis of prospectively collected database | N | Y | Y (All) | 1051 | India | Hospital | Urban | US/Biopsy |
| Chalmers et al. | 2019 | BMJ Open | Cross-sectional | Y | N | N | 2158 | India | Hospital/Community | Urban/Rural | US |
| Choudhary et al. | 2017 | Clinical Transplantation | Retrospective analysis of prospectively collected database | Y | N | N | 573 | India | Hospital | Urban | Biopsy |
| Choudhuri et al. | 2018 | Journal of the Association of the Physicians of India | Cross-sectional | Y | Y | N | 504 | India | Hospital | Urban/Rural | VCTE |
| Das et al. | 2010 | Hepatology | Cross-sectional | Y | N | N | 1911 | India | Community | Rural | US |
| Dassanayake et al. | 2009 | Journal of Gastroenterology and Hepatology | Cross-sectional | Y | N | N | 2985 | Sri Lanka | Community | Urban/Rural | US |
| De et al. | 2023 | International Journal of Obesity | Cross-sectional | N | Y | N | 149 | India | Hospital | Urban | Biopsy |
| Duseja et al. | 2007 | Digestive Diseases and Sciences | Cross-sectional | N | Y | N | 100 | India | Hospital | Urban | US/Biopsy |
| Duseja et al. | 2019 | Journal of Gastroenterology and Hepatology | Cross-sectional | Y | Y | N | 986 | India | Hospital | Urban | US |
| Duseja et al. | 2022 | Metabolic Syndrome and Related Disorders | Cross-sectional | N | Y | Y (Decomp) | 4313 | India | Hospital | Urban | US/Biopsy |
| Goyal et al. | 2020 | Journal of Family Medicine and Primary Care | Cross-sectional | Y | N | N | 200 | India | Hospital | Urban | US |
| Gupta et al. | 2021 | Clinical Nutrition ESPEN | Cross-sectional | Y | Y | N | 250 | India | Hospital | Urban | US/VCTE |
| Gupta et al. | 2023 | Endocrine Regulations | Cross-sectional | N | Y | N | 70 | India | Hospital | Urban | US |
| Gupte et al. | 2004 | Journal of Gastroenterology and Hepatology | Cross-sectional | Y | Y | N | 100 | India | Hospital | Urban | US/Biopsy |
| Hewavisenthi et al. | 2005 | Ceylon Medical Journal | Cross-sectional | Y | N | N | 296 | Sri Lanka | Hospital | Urban | US/Biopsy |
| Jayarama et al. | 2012 | Journal of Clinical and Diagnostic Research | Cross-sectional | Y | N | N | 100 | India | Hospital | Urban | US |
| Kamalraj et al. | 2021 | Diabetes and Metabolic Syndrome: Clinical Research and Reviews | Cross-sectional | Y | Y | N | 1835 | India | Hospital | Urban | US |
| Krishnan et al. | 2011 | Experimental and clinical hepatology | Cross-sectional*^1^ | Y | Y | N | 258 | India | Hospital | Urban | US |
| Kuchay et al. | 2021 | Journal of Gastroenterology and Hepatology | Cross-sectional | Y | Y | N | 531 | India | Hospital | Urban | US/VCTE |
| Kumar et al. | 2022 | Journal of Family Medicine and Primary Care | Cross-sectional | Y | N | N | 190 | India | Hospital | Urban | US |
| Madan et al. | 2004 | Journal of Gastroenterology and Hepatology | Cross-sectional | Y | N | N | 67 | India | Hospital | Urban | Biopsy |
| Madan et al. | 2006 | World Journal of Gastroenterology | Cross-sectional | N | Y | N | 51 | India | Hospital | Urban | Biopsy |
| Majumdar et al. | 2016 | Indian Journal of Public Health | Cross-sectional | Y | Y | N | 176 | India | Community | Rural | US |
| Mishra et al. | 2012 | Indian Journal of Clinical Biochemistry | Cross-sectional | Y | N | N | 645 | India | Hospital | Urban | US |
| Mohan et al. | 2009 | Diabetes Research and Clinical Practice | Cross-sectional | Y | Y | N | 541 | India | Community | Urban | US |
| Niriella et al. | 2019 | Toxic and Metabolic Liver Diseases | Prospective cohort | Y | N | Y (Mortality) | 2985 | Sri Lanka | Community | Urban | US |
| Pinidiyaathirage et al. | 2011 | BMC Research Notes | Cross-sectional | Y | N | N | 401 | Sri Lanka | Community | Rural | US |
| Prasad et al. | 2023 | Indian Journal of Gastroenterology | Cross-sectional | N | Y | N | 272 | India | Hospital | Urban | Biopsy |
| Prashanth et al. | 2009 | The Journal of the Association of Physicians of India | Cross-sectional | Y | Y | N | 83 | India | Hospital | Urban | US/Biopsy |
| Rajput et al. | 2019 | Diabetes and Metabolic Syndrome: Clinical Research and Reviews | Cross-sectional | Y |  |  | 200 | India | Hospital | Urban | US |
| Rastogi et al. | 2022 | Pathology - Research and Practice | Cross-sectional | N | Y | N | 1273 | India | Hospital | Urban | Biopsy |
| Saha et al. | 2017 | Mymensingh Medical Journal | Cross-sectional | Y | N | N | 1019 | Bangladesh | Hospital | Urban | US |
| Sanal et al. | 2011 | Diabetes and Metabolic Syndrome: Clinical Research and Reviews | Cross-sectional | N | Y | N | 76 | India | Hospital | Urban | US/Bloods/Biopsy |
| Sanyal et al. | 2009 | Journal of Indian Medical Association | Cross-sectional | Y | N | N | 470 | India | Hospital | Urban | US |
| Sathiaraj et al. | 2011 | European Journal of Clinical Nutrition | Cross-sectional*^2^ | Y | N | N | 200 | India | Hospital | Urban | US |
| Shah et al. | 2018 | Pakistani Journal of Pharmaceutical Sciences | Cross-sectional | Y | Y | N | 164 | Pakistan | Hospital | Urban | US/Biopsy |
| Sharma et al. | 2017 | Journal of Clinical and Diagnostic Research | Cross-sectional | Y | N | N | 207 | India | Hospital | Not stated | US |
| Shaji et al. | 2022 | Cureus | Cross-sectional | Y | Y | N | 140 | India | Hospital | Urban | Bloods (AST:ALT ratio/FIB 4) and VCTE |
| Singh et al. | 2010 | Indian Journal of Pathology and Microbiology | Cross-sectional | N | Y | N | 60 | India | Hospital | Urban | Biopsy |
| Singh et al. | 2008 | Digestive Diseases and Sciences | Cross-sectional | N | Y | N | 71 | India | Hospital | Urban | Biopsy |
| Singh et al. | 2020 | Polish Journal of Surgery | Prospective observational | Y | Y | N | 101 | India | Hospital | Urban | US |
| Singhai et al. | 2023 | Cureus | Cross-sectional | Y | Y | N | 236 | India | Community | Urban | VCTE |
| Srinivas et al. | 2015 | Indian Journal of Gastroenterology | Cross-sectional | Y | N | N | 1075 | India | Community | Urban | US |
| Taseer et al. | 2009 | Pakistan Journal of Medical Sciences | Cross-sectional | Y | N | N | 100 | Pakistan | Hospital | Urban | US |
| Vanjiappan et al. | 2018 | Diabetes and Metabolic Syndrome: Clinical Research and Reviews | Cross-sectional | Y | Y | N | 300 | India | Hospital | Urban | US |
| Vendhan et al. | 2014 | Diabetes Technology and Therapeutics | Cross-sectional | Y | Y | N | 541 | India | Community | Urban | US |
| Vinyasa et al. | 2022 | Journal of Clinical and Diagnostic Research | Cross-sectional | N | Y | N | 104 | India | Hospital | Urban | US |
| ***1. Initial identification of MASLD cases in diabetic population was used for prevalence (cross-sectional assessment), the study then used MASLD and non-MASLD groups for case-control study.**  ***2. Initial identification of MASLD cases in randomly selected patients attending for health check (cross-sectional assessment), the study then used MASLD and non-MASLD groups for a case-control study**  **Key: MASLD: Metabolic dysfunction associated steatotic liver disease, US: Ultrasound, VCTE: Vibration-controlled transient elastography, AST: Aspartate aminotransferase, ALT: Alanine aminotransferase, FIB4: Fibrosis 4 score, FAST score: Fibroscan-AST score** | | | | | | | | | | | |

**Supplementary Table 2. Breakdown of NIH (National Institute of Health) Quality Assessment Tool scores for included studies. Numerical columns correspond to question in either relevant assessment tool: Observational cohort/cross-sectional studies or case-control studies[16].**

| **Study** | **Year** | **Journal** | **Type of study** | **1** | **2** | **3** | **4** | **5** | **6** | **7** | **8** | **9** | **10** | **11** | **12** | **13** | **14** | **Total** |
| --- | --- | --- | --- | --- | --- | --- | --- | --- | --- | --- | --- | --- | --- | --- | --- | --- | --- | --- |
| Amarapurkar et al. (a) | 2007 | Annals of Hepatology | Cross-sectional | y | n | y | y | n | n | n | n | y | n | y | n | na | n | 5 |
| Amarapurkar et al. (b) | 2007 | Annals of Hepatology | Cross-sectional | y | y | y | n | n | n | n | n | y | n | n | y | na | y | 6 |
| Agarwal et al. | 2011 | Journal of the Association of the Physicians of India | Cross-sectional | y | y | n | y | y | y | y | na | y | n | y | n | na | n | 8 |
| Ajmal et al. | 2014 | Indian Heart Journal | Cross-sectional | y | y | y | y | n | n | n | y | y | n | y | n | na | y | 8 |
| Alam et al. | 2013 | World Journal of Hepatology | Cross-sectional | y | n | y | y | n | n | n | y | y | n | y | n | na | y | 7 |
| Amarapurkar et al. | 2013 | Tropical Gastroenterology | Prospective cohort | y | y | y | y | n | y | y | n | y | n | y | n | y | n | 9 |
| Anand et al. | 2023 | Journal of Clinical and Experimental Hepatology | Cross-sectional | y | y | y | y | y | n | n | y | y | n | y | n | na | y | 9 |
| Anurag et al. | 2015 | Tropical Gastroenterology | Cross-sectional | y | y | y | y | n | n | n | na | n | n | y | n | na | n | 5 |
| Arora et al. | 2022 | European Journal of Clinical Nutrition | Randomised control trial | y | y | y | n | y | y | n | n | y | y | y | n | y | y | 10 |
| Arora et al. | 2023 | Digestive Diseases and Sciences | Cross-sectional | y | y | y | y | n | n | n | n | y | n | y | n | na | y | 7 |
| Banerjee et al. | 2008 | Journal of the Association of the Physicians of India | Cross-sectional | y | y | n | y | y | n | n | y | n | n | y | n | na | n | 6 |
| Baqai et al. | 2023 | Journal of Ayub Medical College Abbottabad | Cross-sectional | y | y | y | y | n | n | n | y | y | n | y | n | na | n | 7 |
| Barik et al. | 2016 | International Journal of Cardiology | Cross-sectional | y | y | n | y | y | n | n | y | y | n | y | n | na | y | 8 |
| Bhat et al. | 2013 | Tropical Gastroenterology | Cross-sectional | y | y | y | y | n | n | n | y | y | n | y | y | na | y | 9 |
| Bhat et al. | 2019 | Sleep and Breathing | Cross-sectional | y | y | y | y | n | n | n | n | y | n | y | y | na | y | 8 |
| Biswas et al. | 2023 | Postgraduate Medical Journal | Retrospective analysis of prospectively collected database | y | y | y | y | n | n | n | y | y | n | y | y | na | y | 9 |
| Chalmers et al. | 2019 | BMJ Open | Cross-sectional | y | y | y | y | y | n | n | y | y | n | y | n | na | y | 9 |
| Choudhary et al. | 2017 | Clinical Transplantation | Retrospective analysis of prospectively collected database | y | y | y | y | n | n | n | y | y | n | y | n | na | n | 7 |
| Choudhuri et al. | 2018 | Journal of the Association of the Physicians of India | Cross-sectional | y | y | y | n | n | n | n | n | y | n | y | n | na | y | 6 |
| Das et al. | 2010 | Hepatology | Cross-sectional | y | y | y | y | n | n | n | y | y | n | y | y | na | y | 9 |
| Dassanayake et al. | 2009 | Journal of Gastroenterology and Hepatology | Cross-sectional | y | y | y | y | n | n | n | n | y | n | y | n | na | y | 7 |
| De et al. | 2023 | International Journal of Obesity | Cross-sectional | y | y | y | y | n | n | n | y | y | n | y | n | na | y | 8 |
| Duseja et al. | 2007 | Digestive Diseases and Sciences | Cross-sectional | y | y | n | y | n | n | n | n | y | n | y | n | na | y | 6 |
| Duseja et al. | 2019 | Journal of Gastroenterology and Hepatology | Cross-sectional | y | y | n | y | n | n | n | y | y | n | y | n | na | y | 7 |
| Duseja et al. | 2022 | Metabolic Syndrome and Related Disorders | Cross-sectional | y | y | y | n | n | n | n | n | y | n | y | n | na | n | 5 |
| Goyal et al. | 2020 | Journal of Family Medicine and Primary Care | Cross-sectional | y | y | y | y | n | n | n | y | y | n | y | n | na | n | 7 |
| Gupta et al. | 2021 | Clinical Nutrition ESPEN | Cross-sectional | y | y | y | y | y | n | n | y | y | n | y | n | na | y | 9 |
| Gupta et al. | 2023 | Endocrine Regulations | Cross-sectional | y | y | y | y | n | n | n | y | y | n | y | y | na | y | 9 |
| Gupte et al. | 2004 | Journal of Gastroenterology and Hepatology | Cross-sectional | y | y | y | y | n | n | n | y | y | n | y | y | na | y | 9 |
| Hewavisenthi et al. | 2005 | Ceylon Medical Journal | Cross-sectional | y | n | n | n | n | n | n | n | y | n | y | n | na | n | 3 |
| Jayarama et al. | 2012 | Journal of Clinical and Diagnostic Research | Cross-sectional | y | y | y | y | n | n | n | n | y | n | y | n | na | y | 7 |
| Kamalraj et al. | 2021 | Diabetes and Metabolic Syndrome: Clinical Research and Reviews | Cross-sectional | y | y | y | y | n | n | n | n | y | y | y | n | na | y | 8 |
| Krishnan et al. | 2011 | Experimental and clinical hepatology | Case control study | y | y | n | y | y | y | y | y | y | n | n | n | na | x | 8 |
| Kuchay et al. | 2021 | Journal of Gastroenterology and Hepatology | Cross-sectional | y | y | y | y | n | n | n | n | y | n | y | n | na | y | 7 |
| Kumar et al. | 2022 | Journal of Family Medicine and Primary Care | Cross-sectional | y | y | y | y | n | n | n | y | y | n | y | n | na | n | 7 |
| Madan et al. | 2004 | Journal of Gastroenterology and Hepatology | Cross-sectional | y | y | y | y | n | n | n | y | y | n | y | n | na | n | 7 |
| Madan et al. | 2006 | World Journal of Gastroenterology | Cross-sectional | y | y | n | y | n | n | n | n | y | n | y | n | na | y | 6 |
| Majumdar et al. | 2016 | Indian Journal of Public Health | Cross-sectional | y | y | y | y | y | n | n | na | n | n | y | n | na | n | 6 |
| Mishra et al. | 2012 | Indian Journal of Clinical Biochemistry | Cross-sectional | y | y | y | y | n | n | n | n | y | n | y | n | na | n | 6 |
| Mohan et al. | 2009 | Diabetes Research and Clinical Practice | Cross-sectional | y | y | y | y | n | n | n | n | y | n | y | y | na | y | 8 |
| Niriella et al. | 2019 | Toxic and Metabolic Liver Diseases | Prospective cohort | y | y | n | y | n | y | n | n | y | n | y | n | y | y | 8 |
| Pinidiyaathirage et al. | 2011 | BMC Research Notes | Cross-sectional | y | y | y | y | n | n | n | n | n | n | y | n | na | y | 6 |
| Prasad et al. | 2023 | Indian Journal of Gastroenterology | Cross-sectional | y | y | y | y | y | n | n | y | y | n | y | n | na | n | 8 |
| Prashanth et al. | 2009 | The Journal of the Association of Physicians of India | Cross-sectional | y | y | n | y | n | n | n | y | y | n | y | n | na | y | 7 |
| Rajput et al. | 2019 | Diabetes and Metabolic Syndrome: Clinical Research and Reviews | Cross-sectional | y | y | y | y | n | n | n | y | y | n | y | n | na | y | 8 |
| Rastogi et al. | 2022 | Pathology - Research and Practice | Cross-sectional | y | y | y | y | n | y | n | y | y | n | y | n | na | n | 8 |
| Saha et al. | 2017 | Mymensingh Medical Journal | Cross-sectional | y | n | n | y | n | n | n | n | y | n | n | n | na | y | 4 |
| Sanal et al. | 2011 | Diabetes and Metabolic Syndrome: Clinical Research and Reviews | Cross-sectional | y | y | y | y | n | n | n | y | y | n | y | n | na | n | 7 |
| Sanyal et al. | 2009 | Journal of Indian Medical Association | Cross-sectional | y | y | y | y | n | n | n | y | y | n | y | n | na | n | 7 |
| Sathiaraj et al. | 2011 | European Journal of Clinical Nutrition | Case control study | y | y | n | y | y | y | y | na | n | n | y | y | na | x | 8 |
| Shah et al. | 2018 | Pakistani Journal of Pharmaceutical Sciences | Cross-sectional | y | y | y | y | n | n | n | n | y | n | y | n | na | y | 7 |
| Sharma et al. | 2017 | Journal of Clinical and Diagnostic Research | Cross-sectional | y | y | y | y | n | n | n | n | y | n | y | n | na | n | 6 |
| Shaji et al. | 2022 | Cureus | Cross-sectional | y | y | n | y | n | n | n | y | y | n | y | y | na | y | 8 |
| Singh et al. | 2010 | Indian Journal of Pathology and Microbiology | Cross-sectional | y | n | n | y | n | n | n | y | y | n | y | n | na | n | 5 |
| Singh et al. | 2008 | Digestive Diseases and Sciences | Cross-sectional | y | y | n | y | n | n | n | y | y | n | y | n | na | n | 6 |
| Singh et al. | 2020 | Polish Journal of Surgery | Cross-sectional | y | y | n | y | n | n | n | n | y | n | y | n | na | n | 5 |
| Singhai et al. | 2023 | Cureus | Cross-sectional | y | y | y | y | n | n | n | y | y | n | y | n | na | y | 8 |
| Srinivas et al. | 2015 | Indian Journal of Gastroenterology | Cross-sectional | y | y | y | y | n | n | n | n | y | n | y | n | na | y | 7 |
| Taseer et al. | 2009 | Pakistan Journal of Medical Sciences | Cross-sectional | y | n | n | y | n | n | n | n | n | n | n | n | na | n | 2 |
| Vanjiappan et al. | 2018 | Diabetes and Metabolic Syndrome: Clinical Research and Reviews | Cross-sectional | y | y | y | y | y | n | n | y | y | n | y | n | na | y | 9 |
| Vendhan et al. | 2014 | Diabetes Technology and Therapeutics | Cross-sectional | y | y | y | y | y | n | n | y | y | n | y | n | na | y | 9 |
| Vinyasa et al. | 2022 | Journal of Clinical and Diagnostic Research | Cross-sectional | y | y | y | y | y | n | n | y | y | n | y | n | na | y | 9 |
